# Virological Surveillance and Molecular Characterization of Human Parainfluenzavirus Infection in Children with Acute Respiratory Illness: Germany, 2015–2019

**DOI:** 10.3390/microorganisms9071508

**Published:** 2021-07-14

**Authors:** Djin-Ye Oh, Barbara Biere, Markus Grenz, Thorsten Wolff, Brunhilde Schweiger, Ralf Dürrwald, Janine Reiche

**Affiliations:** 1 Unit 17, Department of Infectious Diseases, Influenza and Other Respiratory Viruses, National Influenza Centre, Robert Koch Institute, Seestraße 10, D-13353 Berlin, Germany; OhD@rki.de (D.-Y.O.); BiereB@rki.de (B.B.); WolffT@rki.de (T.W.); SchweigerB@rki.de (B.S.); DuerrwaldR@rki.de (R.D.); 2 Consultant Laboratory for RSV, PIV and HMPV, Unit 17, Department of Infectious Diseases, Influenza and Other Respiratory Viruses, Robert Koch Institute, Seestraße 10, D-13353 Berlin, Germany; markusgrenz@gmx.de

**Keywords:** human parainfluenza virus, orthorubulavirus, respirovirus, acute respiratory infection, influenza-like illness, croup, pneumonia, surveillance, molecular epidemiology, phylogeny

## Abstract

Human parainfluenza viruses (HPIVs) are important causes of respiratory illness, especially in young children. However, surveillance for HPIV is rarely performed continuously, and national-level epidemiologic and genetic data are scarce. Within the German sentinel system, to monitor acute respiratory infections (ARI), 4463 respiratory specimens collected from outpatients < 5 years of age between October 2015 and September 2019 were retrospectively screened for HPIV 1–4 using real-time PCR. HPIV was identified in 459 (10%) samples. HPIV-3 was the most common HPIV-type, with 234 detections, followed by HPIV-1 (113), HPIV-4 (61), and HPIV-2 (49). HPIV-3 was more frequently associated with age < 2 years, and HPIV-4 was more frequently associated with pneumonia compared to other HPIV types. HPIV circulation displayed distinct seasonal patterns, which appeared to vary by type. Phylogenetic characterization clustered HPIV-1 in Clades 2 and 3. Reclassification was performed for HPIV-2, provisionally assigning two distinct HPIV-2 groups and six clades, with German HPIV-2s clustering in Clade 2.4. HPIV-3 clustered in C1, C3, C5, and, interestingly, in A. HPIV-4 clustered in Clades 2.1 and 2.2. The results of this study may serve to inform future approaches to diagnose and prevent HPIV infections, which contribute substantially to ARI in young children in Germany.

## 1. Introduction

Human parainfluenzaviruses (HPIVs) are important respiratory pathogens, which are second only to respiratory syncytial virus (RSV) as a cause of hospitalization for respiratory illness among infants and young children [1,2,3,4]. HPIVs are enveloped, negative, single-stranded RNA viruses of the *Paramyxoviridae* family. Their genome has an approximate length of 15,500 nucleotides and encodes six proteins. Of these, the hemagglutinin-neuraminidase (HN) and the fusion (F) protein are surface glycoproteins that are major antigenic determinants [1,5,6]. The *HN* gene, which displays a high degree of genetic variability, has been the basis of most HPIV molecular epidemiology studies to date [7,8,9,10,11].

HPIVs fall into four distinct types, which vary by their genetic and antigenic characteristics. HPIV-1 and -3 belong to the genus *Respirovirus*, whereas HPIV-2 and 4 belong to the genus *Orthorubulavirus* [7,9,12,13,14]. HPIV-1 and -2 are the leading cause of croup, of which HPIV-1 is the most common etiologic agent [15]. HPIV-3, generally considered the most virulent HPIV type, is a frequent cause of bronchiolitis and pneumonia in young infants [16,17,18]. Data on the clinical presentation of HPIV-4 infection are scarce because this HPIV type has been underdiagnosed in the past; the few existing reports indicate an etiologic role in lower respiratory tract illness that may be comparable to that of HPIV-3 [19,20,21]. Seroprevalence data suggest that primary infection with HPIV-3 and -4 occurs earlier in life than infection with HPIV-1 and -2 [22,23]; it is thought that around 50% of children undergo HPIV-3 infection within the first year of life, whereas HPIV-1 and HPIV-2 predominantly affect children 3–5 years of age [15,22,24].

In many cases, HPIV infections lead to mild clinical illness only, presenting with upper respiratory infection (common cold) symptoms that are typically managed in the outpatient setting, where viral etiology is typically not included in the diagnostic workup. This results in an underestimation of the total burden of HPIV infections [3,5,15,25]. HPIV accounts for 20 to 40 percent of viral lower respiratory tract illnesses in children [3,24] and for approximately 7% of hospitalizations for febrile or respiratory illness in those under five years of age [18,26]. It is estimated that HPIV is responsible for approximately 30,000 pediatric hospitalizations annually in the United States alone [26]. In addition, HPIV infections may cause severe illness in adult populations, in particular among the elderly, the immunocompromised, and those with preexisting lung conditions [18,27,28]; between 2 and 12% of adult hospitalizations for respiratory illnesses are attributed to HPIV infection [18]. Thus, the overall burden of HPIV disease is extensive and prevention, especially in young infants, would result in substantial public health benefits [5]. Accordingly, the development of HPIV vaccines has been underway for several years, and some candidates, including an mRNA vaccine, have successfully completed phase 1 clinical trials [5,29,30,31,32]. Given the recent global experience with vaccines against SARS-CoV-2, another respiratory virus, there is great promise that HPIV infection may soon become a vaccine-preventable disease. To ensure an efficient deployment of these vaccines, a detailed understanding of the epidemiological and clinical characteristics of HPIV infection is needed. However, these characteristics vary, not only by HPIV type, but also by region and over time, and there has been a paucity of national-level surveillance data in recent years, especially in Europe. With respect to vaccine efficacy, a crucial factor to consider is pathogen genetic variation, as vaccines covering multiple genetic variants may induce broader, and potentially more robust, protection. Novel technologies facilitate expeditious designing and redesigning of vaccines, thus opening up the possibility to optimally adjust them to pathogen genetic diversity, even in the case of partial immune evasion [33,34]. Therefore, the molecular epidemiology of HPIV types 1–4 circulating in different regions of the world needs to be well characterized. However, the HPIVs circulating worldwide rarely undergo molecular characterization. Phylogenetic analyses are published sporadically only, often in the context of single-location studies and involving only one of the four known HPIV types [10,11,21,35,36,37,38,39,40,41]. As a result, HPIV sequence information available in the public domain is scarce; at the time of this writing, a total of 2633 hemagglutinin-neuraminidase (HN) gene or whole-genome sequences were available at GenBank (HPIV1: 530, HPIV2: 169, HPIV3: 1793, HPIV4: 141; GenBank query version 04/30/2021). Of these, 715 (27%) were full genome sequences. Only seven sequences (HPIV-3) representing partial L-nucleocapsid-associated genes were reported from Germany; however, these open reading frames are not well-suited for phylogenetic analyses.

To characterize, for the first time, the significance of HPIV as a respiratory pathogen in German children at the national scale, we employed our established sentinel surveillance system to assess the frequency, seasonality, and clinical presentation of infections with all four HPIV types. Our results, covering an observation period of four years, confirm previous notions on the epidemiology of HPIV types 1–3 and include the first molecular diagnostic data on the circulation of HPIV type 4 in Germany, which has historically been underestimated. Moreover, we developed a molecular method to sequence the full length of the viral HN gene and performed a comprehensive phylogenetic analysis of each HPIV type, enabling a broader understanding of HPIV genetic diversity, which informs diagnostic strategies and is key to advancing the rational development of safe and effective vaccines.

## 2. Materials and Methods

### 2.1. Patients and Samples

The German sentinel system for monitoring acute respiratory infections (with a focus on influenza) has been established by the National German Working Group on Influenza and includes a long-established laboratory-based virologic surveillance system, which has been previously described [42,43,44,45,46]. Briefly, around 150 sentinel primary care physician practices located in geographically representative areas in Germany were asked to obtain upper respiratory specimens from outpatients presenting with ARI/influenza-like illness (ILI), prioritizing those with indications of systemic illness, e.g., fever. Samples were processed at the Robert Koch Institute, where they undergo molecular diagnostics for respiratory viruses, including influenza viruses A and B (IAV, IBV), RSV, rhinovirus (HRV), adenovirus (AdV), and metapneumovirus (HMPV). To assess the importance of HPIV as a respiratory pathogen in German children, specimens from all sentinel patients under five years of age were selected for retrospective screening with a multiplex real-time PCR specific for HPIV types 1 to 4, spanning an observation period of four years (1 October 2015–30 September 2019).

### 2.2. Ethics Statement

All investigations were conducted according to the principles expressed in the Helsinki Declaration. Written and informed consent was obtained from at least one parent/legal guardian of the child. The German national surveillance of influenza and other respiratory viruses was approved by the Charité-Universitätsmedizin Berlin Ethical Board (Reference EA2/126/11); in addition, sentinel surveillance was covered by German legislation (§4, §13, Protection against Infection Act).

### 2.3. Nomenclature

In recent years, HPIV types (species) 1–4 have been renamed human respirovirus 1 (HPIV-1), human respirovirus 3 (HPIV-3), human orthorubulavirus 2 (HPIV-2), and human orthorubulavirus 4 (HPIV-4) in order to reflect their belonging to the genera *orthorubulavirus* and *respirovirus*, respectively [12,47,48]. Throughout this article, the acronyms HPIV-1–4 are used to maintain consistency with previously published literature.

### 2.4. RT-PCR

Sample processing, RNA extraction, and c-DNA synthesis were performed according to our standard operating procedures, which have been described in considerable detail elsewhere [42,43,45]. HPIV types 1–4 were detected using a quantitative real-time RT-PCR system as recently described [45]. Briefly, we employed a multiplex diagnostic PCR targeting the HN gene region of HPIV types 1–3 and the P-gene of HPIV types 4. One reaction contained, in a total volume of 20 µL, 1× PCR buffer, 4 mmol/L MgCl_2_, 1 mmol/L deoxynucleoside triphosphate (dNTP; Thermo Fisher Scientific, Waltham, MA, USA) with deoxyuridine triphosphate (dUTP; GE Healthcare, Chicago, IL, USA), 600 ng of bovine serum albumin (BSA; Thermo Fisher Scientific, Waltham, MA, USA), 0.3 U (singleplex) or 1 U (multiplex) Platinum Taq Polymerase (Thermo Fisher Scientific, Waltham, MA, USA), 5 µL of prediluted c-DNA, and the oligonucleotides listed in Table S1 (Metabion, Planegg, Germany and Applied Biosystems, Foster City, CA, USA). PCR was performed in 96-well plates on an LC480II real-time PCR thermal cycler (Roche, Basel, Switzerland), using the following thermocycling parameters: 95 °C for 5 min, followed by 45 cycles of 95 °C for 15 s and 60 °C for 30 s. In addition to HPIV-PCR, all samples underwent molecular testing for IAV, IBV, RSV, HRV, AdV, and HMPV, using RT-PCR protocols that have been described in considerable detail elsewhere [45,49].

### 2.5. HPIV Sequencing

Of at least 20% of HPIV-positive samples, the HN protein gene was amplified by nested PCR spanning the complete HN coding sequence. The first amplification was performed with 2.5 µL of c-DNA in a 25 µL reaction by using 300 nM of sense and antisense primer, respectively, for each HPIV type (Metabion, Planegg, Germany), 100 µM deoxynucleoside triphosphates, 2 mM MgCl_2_, 1 U Platinum Taq DNA polymerase (Invitrogen, Karlsruhe, Germany), and PCR buffer (200 mM Tris-HCl (pH 8.4), 500 mM KCl). Amplification was carried out at 95 °C for 5 min, followed by 10 cycles at 94 °C for 30 s, TA °C (Table 1) for 30 s, and 72 °C for 90 s, and by 20 cycles at 94 °C for 30 s, TA °C for 30 s, and 72 °C for 90 s + 5 s per cycle, with a final extension at 72 °C for 10 min. A 2 µL volume of a 1:100 dilution (1:1000 for HPIV-3) of the external PCR reaction was used for nested PCR, which was performed in a 50 µL reaction with the same reagents and concentrations as above. The cycling protocol was 94 °C for 5 min, followed by 40 cycles at 94 °C for 30 s, TA °C (annealing temperature, see Table 1) for 30 s, and 72 °C for 60 s, with a final extension at 72 °C for 10 min. The nested amplicons were visualized by agarose gel electrophoresis. The PCR products were purified either directly with MSB Spin PCRapace or Invisorb Spin DNA Extraction Kit (Stratec Molecular, Berlin, Germany) according to the manufacturer’s instructions. Purified PCR products were cycle sequenced in the forward and the reverse directions with primer pairs previously used for nested PCR.

Prior to sequencing, HPIV-positive samples were differentiated into types 4a and 4b, respectively. To this end, 3 µL of c-DNA was amplified in a 50 µL reaction by using 300 nM of each sense and antisense primer (Metabion, Planegg, Germany), 100 µM deoxynucleoside triphosphates, 2 mM MgCl_2_, 1 U Platinum Taq DNA polymerase (Invitrogen, Karlsruhe, Germany), and PCR buffer (200 mM Tris-HCl (pH 8.4), 500 mM KCl). Amplification was carried out at 94 °C for 5 min, followed by 45 cycles at 94 °C for 30 s, 55 °C for 30 s, and 72 °C for 30 s, with a final extension at 72 °C for 10 min. PCR products were visualized by agarose gel electrophoresis.

### 2.6. Sequence Analyses

For each HPIV type, HN nucleotide sequences were aligned with reference sequences from human clinical samples retrieved from GenBank using the MAFFT algorithm [50,51] in Geneious Prime^®^ 2020.2.3. Each dataset was trimmed to produce the same length for all sequences within the alignments, in particular HPIV-1 with 1613nt (Reference Strain JQ901976, ORF: 6843–8455), HPIV-2 with 1688nt (Reference Strain AF533012, ORF: 6821–8508), HPIV-3 with 1619nt (Reference Strain JN089924, ORF: 1–1619), and HPIV-4 with 1737nt (Reference Strain EU627591, ORF: 7563–9299). Sequences containing “N” or ambiguous nucleotides were removed from the alignment, as were identical nucleotide sequences detected with DNAcollapser (https://users-birc.au.dk/palle/php/fabox/dnacollapser.php, accessed on 1 June 2021). Only one representative nonidentical sequence was kept per epidemic season/year and country.

Maximum likelihood trees were calculated with the best-fit substitution model General Time Reversible with Gamma distribution (GTR+G) for HPIV-1, the Tamura 3-parameter model with Gamma distribution (TN92+G) for HPIV-2, the Tamura-Nei model with Gamma distribution and invariant sites (GTR+G+I) for HPIV-3, and the Tamura 3-parameter model with Gamma distribution and invariant sites (T92+G+I) for HPIV-4, respectively, in MEGA version X [52]. The reliability of the branching order was estimated by performing 1000 bootstrap replicates, with values ≥ 80 defined as well supported. The trees were manually edited in CorelDRAW version X6.

The mean genetic distances within and between clades were calculated using MEGA version X [52] with the most simplified method, the *p*-distance, and pairwise deletion for alignments with gaps. Distances were described in terms of mean and standard error by the bootstrap method with 1000 replicates.

The clade definition was adapted from Goya et al. [2]. A clade was defined as a monophyletic cluster with high statistical support (≥80% bootstrap value) that comprised ≥3 epidemiologically unrelated sequences, i.e., nonidentical sequences from different countries and/or outbreaks (epidemic season) [2]. Small numbers of independent sequences not fitting the clade definition were not classified and will remain undefined until future sequences will cluster with them so that the clade definition is met [2]. In order to ascertain that the *p*-distance between clades is higher than the distance within clades, the highest mean intraclade *p*-distance was used as the threshold for sorting viruses into a different clade. This clade definition procedure was also applied to define groups of HPIV-2 and HPIV-4.

### 2.7. Nucleotide Sequence Accession Numbers

The GenBank accession numbers of the HN nucleotide sequences obtained in the present study are MW654384–MW654416 and MW654434–MW654449 for HPIV-1, MW654450–MW654480 for HPIV-2, MW645230–MW645237 and MW645252–MW654383 for HPIV-3, and MW645220–MW645229 for HPIV-4.

### 2.8. Statistical Analysis

The nonparametric Mann–Whitney U-test was used to assess the statistical significance of age differences between the total study population and subgroups of children infected with different HPIV types. The χ^2^ test was employed to compare HPIV-infected children to the total study population with respect to clinical presentation.

### 2.9. Graphics

Graphics were created using the Excel Maps function in Microsoft version 365 and GraphPad Prism Version 9.

## 3. Results

### 3.1. Demographics of the Study Population

**Figure 1:**
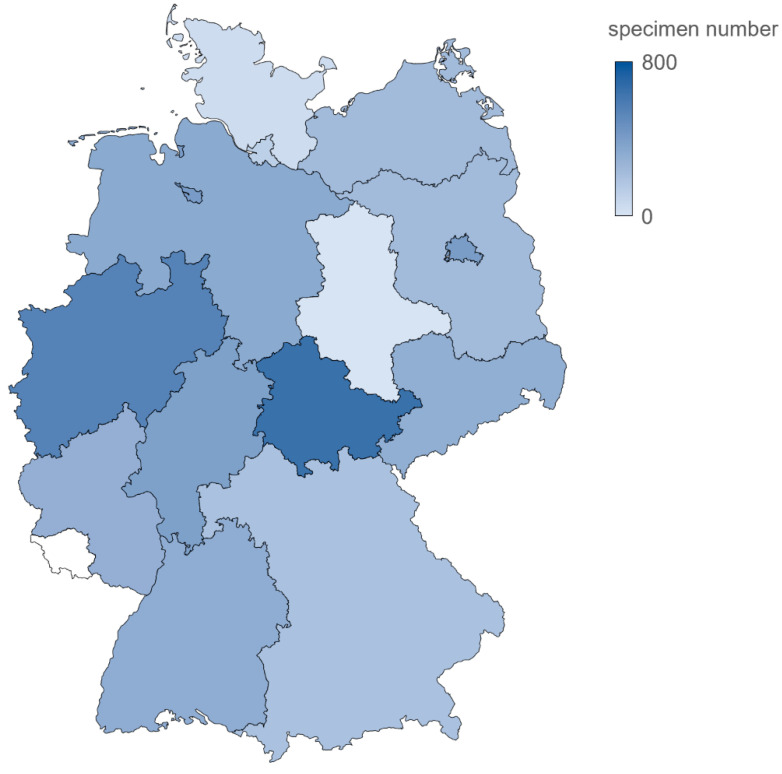
Distribution of specimens collected in Germany, 10/1/2015–09/30/2019. Sentinel practices were distributed over 15 of the 16 German Bundesländer (Federal States). Color codes represent, for each *Bundesland* (Federal State), the number of specimens collected.

A total of 4463 respiratory specimens, collected from pediatric patients presenting with ARI between 1 October 2015 and 30 September 2019, were included in this study. Of these, 896 samples were obtained in the season 2015/16 (1 October 2015–30 September 2016), 1178 in 2016/17, 1279 in 2017/18, and 1110 in 2018/19. Within the total study population, 2081 (46.6%) were female, and 2363 (52.9%) were male; median age was 24 months (interquartile range, IQR: 14–36 months), and the majority (69.9%) were under 2 years of age. Patients were from 15 *Bundesländer* (Federal states) (Table 2 and Figure 1).

### 3.2. Demographic Information on HPIV-Positive Specimens and Coinfections with Other Respiratory Viruses

Of the 4463 samples analyzed, 459 (10.3%) were positive for HPIV types 1–4. HPIV-1–4 was detected in every season, at annual frequencies that appeared to follow a weak biennial pattern. Seasons with a relatively low HPIV frequency alternated with seasons displaying relatively high HPIV frequency, i.e., 2015/16, where HPIV detected in 8.4% samples was followed by 2016/17 (11.5%) and 2017/18 (9.3%) was followed by 2018/19 (11.7%; Table 3). Among the HPIV-positive specimens, 250 (54.5%) were from male patients. HPIV-infected children had a median age of 22 (IQR: 15–24) months, and the majority (356 or 77.6%) were ≤2 years of age. Age and gender distributions did not differ substantially between seasons (Table 3).

Coinfections with other respiratory viruses were detected in 125 of the HPIV-positive specimens (Figure S1). One coinfecting pathogen was found in 112 specimens, the most common being HRV (*n* = 68), followed by AdV (*n* = 19), RSV (*n* = 11), IAV/IBV (*n* = 9), and HMPV (*n* = 5). Two coinfecting pathogens were found in twelve specimens, of which eleven displayed AdV with another virus, and one displayed HRV with RSV. Three coinfecting pathogens (AdV, HRV, and RSV) were present in one specimen.

### 3.3. Clinical Presentation

The most common symptoms among HPIV-positive children were rhinorrhea (93.7%), cough (91.1%), and fever/chills (90.0%) (Table 4). Precisely 81.9% presented with ILI, defined as illness of sudden onset featuring at least one systemic symptom (fever, cephalgia, or myalgia) and at least one respiratory symptom (cough or pharyngitis). Cough was significantly more frequent in HPIV-infected children than in the total study population, especially among children with HPIV-1 and HPIV-2. In children with HPIV-3, rhinorrhea was slightly more common, whereas myalgias/cephalgias and pneumonia were less frequently observed than in the total study populations. It is worth noting that myalgias/cephalgias are difficult to confirm in small children, and therefore, this association has to be interpreted with caution. In children infected with HPIV-4, the frequencies of fever/chills (73.8%) and ILI (60.7%) were significantly lower, and the frequency of pneumonia (13.1%) was significantly higher than in the total study population (Table 4). On average, children with HPIV-1 infection were significantly older (28 months), and children with HPIV-3 infection were significantly younger (20 months) than the total study population (24 months).

### 3.4. Incidence of HPIV Types 1–4

From 2015/16 to 2018/19, a single HPIV type was identified in 457 of the 459 HPIV-positive samples. HPIV-3 was detected in 234 (51% of HPIV-positive) specimens, thus representing the most common HPIV type (Figure S2A). HPIV-1 was present in 113 (25%) samples, HPIV-4 in 61 (13%) samples, and HPIV-2 was found in 49 (11%) samples. Of the two specimens with evidence for coinfection, one was positive for both HPIV-3 and HPIV-2, and the other was positive for HPIV-3 and HPIV-4.

### 3.5. Seasonality

All HPIV types 1 to 4 were detected in every season (year), albeit at varying frequencies that appeared to be influenced by type. Thus, the type composition of HPIV-positive samples varied by season, as shown in Figure S2A,B.

**Figure 2:**
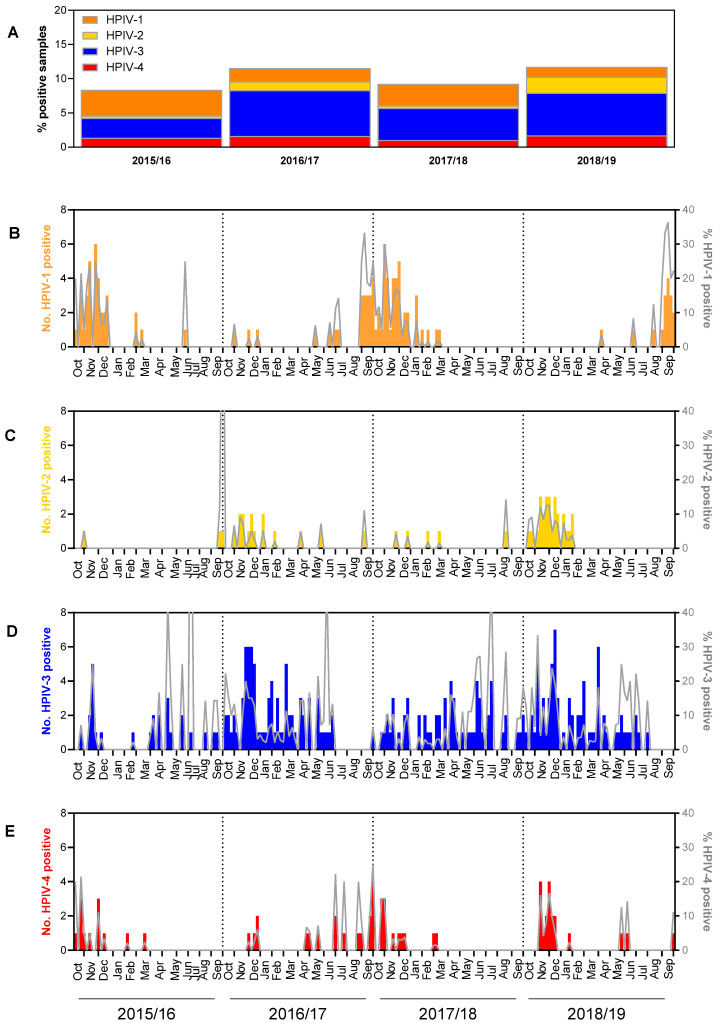
(**A**) Percentage of HPIV-positive samples per season and distribution of HPIV types among them, 2015/16–2018/19. (**B**–**E**) Weekly numbers and percentages of samples positive for HPIV types 1–4. Bar plots indicate the number of positive samples and are scaled by the left *y*-axis, whereas each line represents the percentage of positive samples and is scaled by the right *y*-axis.

HPIV-1 and, to a lesser extent, HPIV-4 displayed higher frequencies in odd years (i.e., seasons starting with an odd year: 2015/16 and 2017/18) (HPIV-1: 35–45%; HPIV-4: 10–16%) than in even years (i.e., seasons starting with an even year: 2016/17 and 2018/19) (HPIV-1: 12–17%; HPIV-4: 10–15%). By contrast, HPIV-2 and HPIV-3 frequencies were relatively high in even years (HPIV-2: 11–20%; HPIV-3: 53–59%) and relatively low during odd years (HPIV-2: 4%; HPIV-3: 34–50%). These biennial circulation patterns were pronounced for HPIV-1, HPIV-2, and HPIV-3, while they were more subtle for HPIV-4 (Supplementary Figure S1B). Similarly, the overall HPIV detection frequency varied by seasonal year, reflecting predominantly variations in the detection of HPIV-3, the most common subtype (Figure 2A).

HPIV-1 detections peaked primarily in autumn/early winter of odd years, i.e., October–December, 2015; September, 2017–January, 2018; and September, 2019. In addition, this HPIV type was sporadically detected during the summer months (Figure 2B). HPIV-2 detections were observed to be highest between October and January during the 2016/17 and 2018/19 seasons (Figure 2C). HPIV-3 displayed clear peaks in November/December 2016 and 2018, but it was also present in samples obtained from January to June 2017, January to October 2018, and January to June 2019 (Figure 2D). HPIV-4 detections rates were highest in autumn, e.g., in October/November 2015, 2017, and 2018 (Figure 2E). Regardless of type, HPIV detection rates generally dropped from January to March, i.e., during the time period that typically defines the German influenza season.

### 3.6. Molecular Characterization

**Figure 3:**
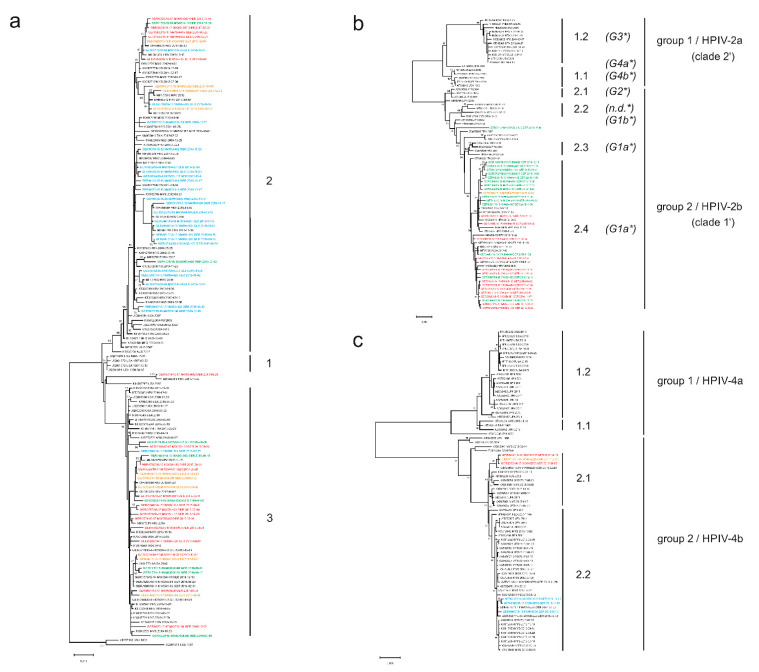

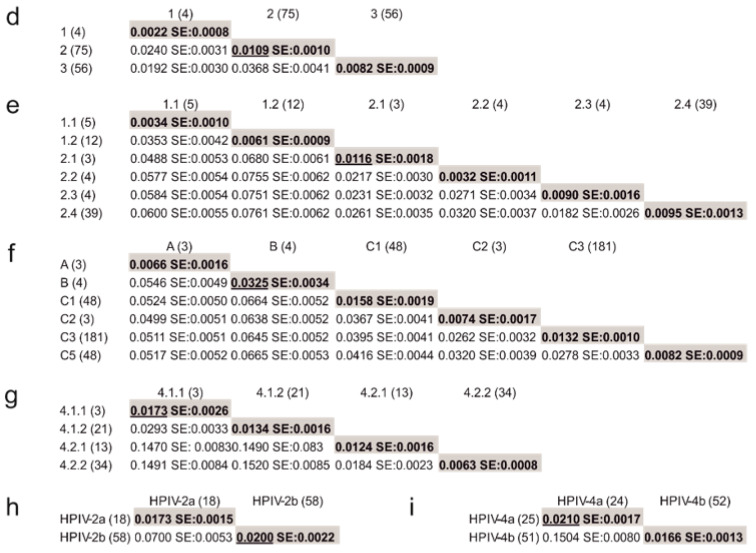
Phylogenetic tree analyses and estimates of average genetic *p*-distances within and between HPIV clades. Maximum likelihood trees of the partial HN gene are shown for HPIV-1 (**a**), HPIV-2 (**b**), and HPIV-4 (**c**). German sequences of this study are colored by epidemic season: 2015/16 in blue, 2016/17 in red, 2017/18 in orange, and 2018/19 in green. Clades are indicated on the right. Only bootstrap values ≥80% are displayed at the branch nodes. Genetic *p*-distances were calculated for genetic clades of HPIV-1 (**d**), HPIV-2 (**e**), HPIV-3 (**f**), and HPIV-4 (**g**), as well as for genetic groups of HPIV-2 (**h**) and HPIV-4 (**i**). The number of sequences of each clade or group is included in parenthesis. *p*-distances within each clade or group are shown with a gray background. The highest mean intraclade/intragroup *p*-distance is underlined. The standard error (SE) is shown next to the mean *p*-distance.* clades defined by [38]; ‘ clades defined by [53].

**Figure 4:**
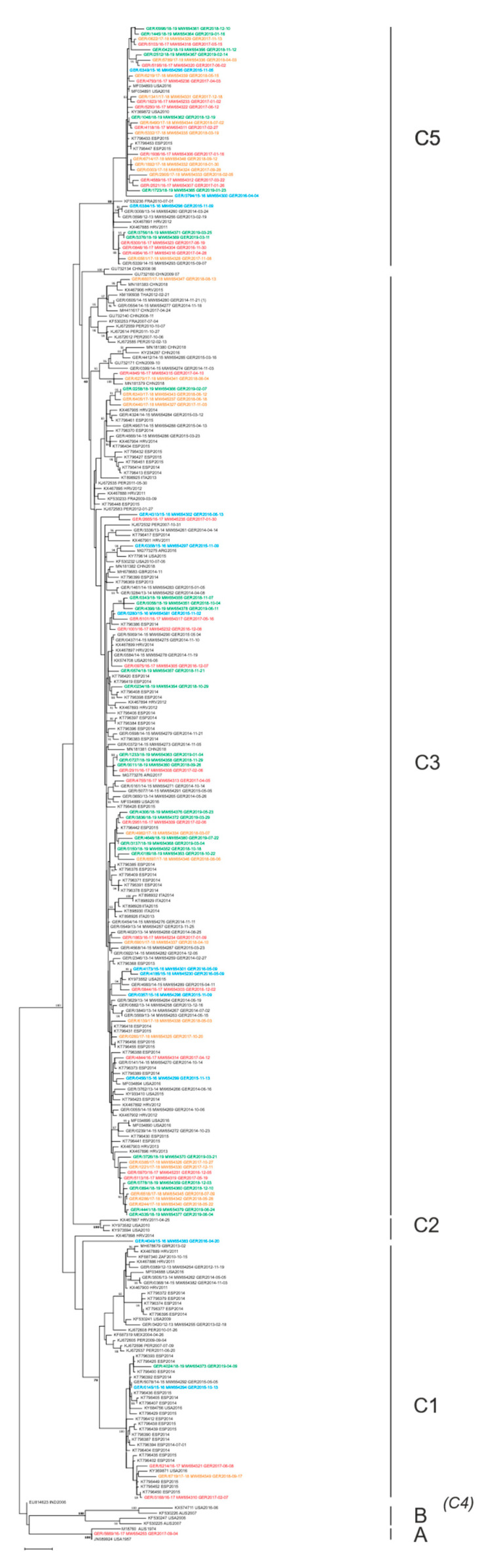
Maximum likelihood tree of the partial HN gene of HPIV-3. German sequences of this study are colored by epidemic season: 2015/16 in blue, 2016/17 in red, 2017/18 in orange, and 2018/19 in green. Clades are indicated on the right. Only bootstrap values ≥ 80% are displayed at the branch nodes.

To characterize HPIV strains circulating in Germany at the molecular level, selected HPIV-positive samples underwent full-length sequencing of the HN gene. The total 191 HPIV type 1–4 specimens with sequencing successfully completed were included in phylogenetic analyses of the HN gene for HPIV types 1–4. Clade assignment was carried out based on the classification of monophyletic clusters with high statistical support (≥80% bootstrap value) and their mean genetic *p*-distance (Figure 3 and Figure 4).

Our phylogenetic analysis demonstrated that HPIV-1 clustered into three different clades, of which Clade 1 seems to be extinct since the year 2000. German HPIV-1 of the seasons 2015/16 to 2018/19 cocirculated in Clades 2 and 3 (Table 5, Figure 3a; for a high-resolution image, see Figure S3).

HPIV-2 sequence information is very limited worldwide, which complicates phylogenetic clade assignment for this HPIV type. However, phylogenetic tree analysis and genetic analysis of the *p*-distance performed in this study provide evidence for the existence of two HPIV-2 groups, based on the criteria applied (Figure 3b,h; for a high-resolution image, see Figure S4). These two HPIV-2 groups were provisionally assigned as Group 1 (HPIV-2a) and Group 2 (HPIV-2b), respectively. Clade designations were adjusted accordingly, resulting in six clades, which were provisionally designated as Clades 1.1 to 1.2 (constituting Group 1) and as Clades 2.1 to 2.4 (constituting Group 2), respectively. German HPIV-2 clustered almost exclusively in Clade 2.4 (Table 5). One sequence, of the 2018/19 season, remained unclassified.

For HPIV-4, phylogenetic analysis of publicly available sequences indicated that each of the two HPIV-4 groups (termed Group 1, which corresponds to HPIV-4a and Group 2, which corresponds to HPIV-4b) comprises two clades (Figure 3c; for a high-resolution image, see Figure S5). HN gene sequencing was completed over the entire length of the HN gene for six German HPIV-4 viruses, which belonged to Clade 2.1 and Clade 2.2 (Table 5).

For HPIV-3, our maximum likelihood analysis confirms the presence of the three established clusters A, B, and C, as well as of the subclusters C1–3 and C5 (Figure 4; for a high-resolution image, see Figure S6) [9,38]. One sequence (EU814623) of the previously defined subcluster C4 [9] remains unclassified in this study due to our classification criteria. Clade definitions within these groups were based on our standard approach, where mean intergenetic *p*-distances exceed the highest mean intragenetic *p*-distance. In this context, Cluster B intragenetic *p*-distances were disregarded because this cluster consisted of few, highly divergent virus sequences and, more importantly, all intergenetic *p*-distances are several times higher than all other intragenetic *p*-distances.

The vast majority of German HPIV-3 sequences were categorized into subclusters C3 and C5 at equal proportions. In addition, 5 HPIV-3 were assigned to Subcluster C1, one virus was assigned to subcluster A, and one could not be assigned to any HPIV-3 subcluster (Table 5, Figure 4). Interestingly, the HN gene of the single German HPIV-3 (GER/5669/16-17) assigned to subcluster A was identical to the HN gene of an HPIV-3 isolated in 1957 in the United States.

## 4. Discussion

Here we report the epidemiological profile of HPIV in pediatric outpatients under five years of age who presented with ARI/ILI symptoms during a 4-year period of national sentinel surveillance. Out of a total of 4463 specimens analyzed, we determined an overall prevalence of 10.3%, ranging from 8.4% in the least to 11.7% in the most active season. These findings confirm the importance of HPIV as a respiratory pathogen in young children. In any study, HPIV detection rates are influenced by the detection method used, by temporal factors, i.e., the season surveyed and duration of the study, and by demographic and clinical features, including the study enrollment criteria. The data reported here are well in line with results from other surveillance studies focusing on pediatric outpatients with ARI/ILI. For example, among outpatients < 18 years with ILI studied over a 5-year period in the United States, an HPIV test prevalence of 9% was found [54], and 8% of pediatric outpatients < 5 years with ARI surveyed in Beijing, China, were positive for HPIV 1–4 [55]. These detection rates, as ours, are higher than those reported by other investigators studying outpatients with ILI. Reed et al. detected HPIV in 5.6% of samples from US American children < 5 years [3], and Villaran et al. reported HPIV in 3.2% samples from ILI patients surveyed in multiple Latin American countries ^7^. However, both of these studies were based on viral cultures, a detection method that is less sensitive than RT-PCR [14,56,57,58]. In addition, Villaran et al. included young adult patients, in whom symptomatic HPIV infection rates are intrinsically lower [7]. Whereas the majority of HPIV-positive samples were negative for other respiratory viruses, viral coinfections were detected in a portion of our study collective. The most common coinfecting pathogen was HRV, as described by other investigators [59,60]. With respect to the prevalence of the different HPIV types, the majority of our HPIV-patients were infected with HPIV-3 (51%) and HPIV-1 (25%), whereas HPIV-4 (13%) and HPIV-2 (11%) were less common. These results are not entirely unexpected. HPIV-3 has been identified as the most common HPIV type in multiple investigations performed to date [22,54,55,61,62,63], and HPIV-1 has frequently been found to be the second most common HPIV type [22,54,55,62]. HPIV-2 is often considered the least frequent HPIV type [22,63]. HPIV-4 has been considered rare in the past, in part due to underdiagnosis because it was more difficult to isolate and type-specific molecular methods were lacking [22,54,64]. Others have found HPIV-4 to represent between 8% and 22% of HPIV-positive samples and given that the frequency of detection for each type is subject to annual fluctuations, the frequencies determined for our study population match these findings well [23,53,55,63].

HPIV is a common cause of acute respiratory infection in infants and young children, whose immune system is naïve to this agent [17,65]. Accordingly, in our study population of pediatric outpatients < 5 years old with ARI/ILI, HPIV was found in all age groups, but it predominantly affected those under two years of age, who were particularly overrepresented among those infected with HPIV-3 and HPIV-4. Thus, the age distribution varied by HPIV type; children diagnosed with HPIV-3 and HPIV-4 infection were younger on average than children diagnosed with HPIV-1 or HPIV-2 infection. For HPIV-3, this observation matches numerous published reports from both inpatient and outpatient settings demonstrating that infection with this most common HPIV type occurs at an earlier age than infections with HPIV types 1 and 2 [3,15,54,66,67]. For HPIV-4, comparable data are scarce because this HPIV type is infrequently recognized; however, the results of a seroprevalence study are consistent with our findings, indicating that HPIV-4 infection does indeed occur earlier in life than HPIV-1/-2 infection [23]. If confirmed by others, these findings might be of interest with respect to potential future vaccine schedules: A reasonable strategy could be to administer not only HPIV-3 but also HPIV-4 vaccines well before HPIV-1/-2 vaccines. As expected, we did not observe an influence of the patient’s gender on detection frequency.

With respect to clinical presentation, there was some variation between HPIV types in our study population. Cough appeared to be a more common presenting symptom in HPIV-1/HPIV-2 infected patients than in those infected with other HPIV types or negative for HPIV. This observation was expected, given that both HPIV-1 and HPIV-2 are frequent causes of croup (laryngotracheobronchitis), an illness whose hallmark is a characteristic cough. In HPIV-4 patients, ILI was rarer, whereas pneumonia was more frequent than in patients infected with other HPIV types or negative for HPIV. This observation confirms a previous report revealing pneumonia in a significant portion of HPIV-4 patients (9 of 34 hospitalized individuals) [19,68]. Despite high seroprevalences, this virus has traditionally been regarded as an uncommon or low-virulence pathogen, because it was rarely cultured from respiratory tract samples. With increasing use of molecular diagnostics in recent years, there has been mounting evidence that HPIV-4 may actually play an important role in the pathogenesis of ARI [19,20,64,68]. It is possible that this virus has been underdiagnosed in the past, due to (i) its fastidious and slow growth in cultures, (ii) a CPE that may not always be present and take weeks to appear, and (iii) the lack of HPIV-4 detection capabilities in antigen-based diagnostic panels [19,64]. Our results render further support to the notion that the clinical severity of HPIV-4 infection is on par with that of PIV-3 infection [20] and further studies are needed to fully assess the importance of HPIV-4 infection.

During the time period of four consecutive years studied here, HPIV type 1–4 detection rates varied by season, following distinct type-specific seasonal profiles. HPIV-1 was detected predominantly in the fall and early winter of odd years, a finding that is consistent with other reports from temperate regions all over the world, such as the United States, Japan, Argentina, and Australia [22,23,54,69,70,71]. By contrast, HPIV-2 circulation, also at its top levels in fall and early winter, was higher in even years than in odd years, as has been reported for the United States and Beijing, China [22,54,55]. HPIV-3 activity appeared to be present almost year-round with detection peaks in November and December and high detection rates in spring and early summer. While this most common HPIV type was detected every year in our sentinel, detections in even years did exceed those in odd years. This two-year periodicity, less pronounced for HPIV-3 than for HPIV-1 or HPIV-2, has previously been noted in virological surveillance data from the United States [22]. HPIV-4 detections, though not particularly common, appeared to also be of cyclic nature, occurring more frequently in the fall and in odd years, with the exception of the 2018/19 season. Our findings are consistent with those of Frost et al. [20], who described year-round PIV-4 prevalence with peaks in the fall of odd-numbered years. Peaks in the fall have been described for this HPIV type previously [2,13,23,63,64], and one group of investigators also commented on the biennial circulation pattern of HPIV-4 [64]. Given that HPIV-4 has not been detected in many studies, our investigations help to address the paucity of seasonality data on this HPIV type. The temporal dynamics we observed imply that HPIV-1 and HPIV-4 circulate concurrently and reach their highest activity levels in odd years, and that they alternate with HPIV-2 and HPIV-3, who also cocirculate and reach their highest activity in levels in even years. Potential causes for this alternating circulation pattern, which appears most pronounced for HPIV-1 and HPIV-3, include viral interference or a certain level of immune cross-protection between HPIV-1/HPIV-4 and HPIV-2/HPIV-3, which might diminish the spread of one HPIV type due to population immunity having been boosted by the other type [22]. During the 2019/2020 season, which has not been included in this analysis, broad nonpharmaceutical interventions related to the COVID-19 pandemic led to a pronounced suppression of all respiratory viruses, including HPIV, in our sentinel [45]. Further studies are needed to assess whether the impact of that season has affected HPIV circulation patterns in the longer term, e.g., by leading to an excessive rebound, similar as observed for HRV [45,72] or RSV [73].

Ours is the first study to provide HN gene sequences including phylogenetic characterization for HPIV types 1–4 from Germany, and, to our knowledge, it is one of only five studies providing HN gene sequence data from HPIV circulating in Europe [10,11,74,75].

HPIV-1 is generally subdivided into three different clades [41]. However, sequencing studies from both different regions of the world and years of sample collection, indicate that HPIV-1 strains circulating since 2003 include Clade 2 and 3 viruses only. Clade 2 included viruses from Australia, France, Mexico, and the USA collected in 2003–2009, whereas Clade 3 included mostly viruses from the USA, and a few sequences from France and South Africa, collected in 2009–2015 [74]. Clades 2 and 3 were found to cocirculate in the USA in 2009 [74]. HPIV-1 obtained from pediatric in- and outpatients in Vietnam between 2009 and 2010 clustered in Clade 2 (corresponding to HPIV-1 Clade 3) [53]. In Europe, cocirculation of Clade 2 and 3 viruses was observed between 2011 and 2014 among Croatian hospitalized patients [76]. In our study of pediatric outpatients in Germany, maximum likelihood analysis demonstrated cocirculation of HPIV-1 Clades 2 and 3 from 2015/16 onwards during the study period. Although sequence data for HPIV-1 are limited, our phylogeny study demonstrates continuous cocirculation of HPIV-1 Clades 2 and 3 from 2003 to 2019 and, in addition, the global disappearance of Clade 1 after 1999, which has previously been observed by others [11,41,53,74,76] (this study). Whereas it appears reasonable to assume that the lineage has died out because Clade 1 viruses have been absent from all analyses for over 20 years, e.g., due to decreased evolutionary fitness, it is also possible that Clade 1 viruses are exceedingly rare and have simply not been detected due to the general scarcity of HPIV-1 sequencing studies [74].

For HPIV-2, two distinct genetic and antigenic clusters were first described in 2008 by Terrier et al. [77]. Briefly afterwards, a phylogenetic tree for HPIV-2 was constructed, which included additional HN gene sequences and suggested the existence of four HPIV-2 clusters (G1–4) [38]. Clusters G1 and G2 constituted the first cluster identified by Terrier et al., while Clusters G3 and G4 constituted the second cluster identified by Terrier et al., respectively. The molecular characterization of additional HPIV-2 isolates collected between 2011 and 2017 led to the determination of more HPIV-2 sequences, which were categorized into the predefined clusters G1 and G3 [10,39]. Cross-neutralization activity between Clusters G1 and G3 was low, indicating the existence of distinct genotype-specific antigenic determinants [10]. The differences in cross-neutralization activity between Clusters G4b and G1a (Clades 1.1 and 2.3 in this study) observed by Terrier et al. and between Clusters G3 and G1a (Clades 1.2 and 2.4 in this study) described by Santak et al. may indicate the existence of two distinct antigenic HPIV-2 groups [10,77]. In line with this, antigenic diversity between different HPIV-2 isolates was described by two other groups [78,79]. Intratype antigenic diversity is not uncommon for HPIV. Differences in tissue-culture neutralization and hemadsorption inhibition have also been observed for HPIV-4 strains; subsequently, the two antigenically distinct groups were assigned to Subtypes HPIV-4a and 4b, respectively [80]. In our study, phylogenetic analysis identified two genetic HPIV-2 groups, characterized by two distinct monophyletic clusters and their corresponding *p*-distances, respectively (Figure 2). Both HPIV-2 groups identified here were provisionally assigned to groups 1 and 2 and correspond to clusters 1 and 2, as originally described by Terrier et al. [77]. Based on the two new assigned groups, clade classification was adapted following the same criteria, and six clades were provisionally assigned as 1.1 and 1.2 belonging to group 1 and as 2.1 to 2.4, respectively, linked to group 2. New defined clades are distinct from the suggested clades defined by Almajhdi et al. [38] (Figure 3). Due to the still low number of HPIV-2 sequences, it might be possible that further adaption of this nomenclature is necessary in the future. A recent study about HPIV-2 in hospitalized patients from Croatia revealed that the 2014–2017 isolates belonged primarily to clade G1a (2.4 in this study), while isolates from 2011 to 2012 belonged predominantly to Clade G3 (1.2 in this study), with G3 (1.2) viruses circulating only sporadically in clade G3 after 2014 [10]. Interestingly, German HPIV-2 viruses from the seasons 2016/17 to 2018/19 clustered exclusively in G3 (2.4. in this study). The observed replacement of clade G3 (2.4. in this study) by G1a (2.4 in this study) in Croatia might be caused by an increase in specific antibodies against G3 in the population, and incomplete immune response against other new emerging HPIV, e.g., G1a [10].

Globally, few sequence data are available, which cover the partial or complete HN gene of HPIV-4. In our study, six HN gene sequences were obtained for HPIV-4b, which belonged to clade 2.2 in the season 2015/16 and to clade 2.1 in the seasons 2016/17 and 2017/18. German HPIV-4b from 2015/16 cluster together with virus sequences from Malaysia isolated in 2012 or 2013 and with a Japanese sequence from 2012. In contrast, German sequences of the seasons 2016/17 and 2017/18 cluster apart from other viruses in clade 2.1, which have been isolated before 2013, indicating that the German cluster demonstrates an ongoing evolution. Given the dearth of publicly available HPIV-2 and -4 sequences, the results of clade assignments for these two HPIV types should be interpreted with caution, as additional sequences could change tree topology in the future.

Phylogenetic papers divide HPIV-3 into clusters A, B, and C, and into subclusters C1–C5 [2,9,11,38]. Further refinements also define lineages within clusters C1 and C3 [2,9,11,38]. In our study, the established classification could be confirmed for clusters and subclusters only and was consequently used for characterization. From 2015/16 to 2018/19, German HPIV-3 sequences belonged to cluster C within the subclades C1, C3, and C5. Over the study period, subcluster C3 was predominant, slightly followed by C5. subclade C1 was continuously cocirculating, but to a lesser extent. Through recent years, Cluster C was circulating globally, and Subclusters C1, C3, and C5 cocirculated in many countries. Whereas for C3, a continuous circulation was observed [2,11,35,36,37], circulation of C1 and C5 appeared more sporadic in some regions, such as Israel, Croatia, and Argentina [2,11,35]. As for Germany, subcluster C1 was detected throughout the whole study period in Japan (2013–2015) and Croatia (2011–2015) [11,36]. Phylogenetic analyses revealed that cluster B contains viruses isolated between 1974 and 2007, mostly in the United States and Australia, while Cluster A comprises HPIV-3 prototype strain and an Australian strain isolated in 1973 [37]. Of note, we identified one HPIV-3 from 2017 with an HN-gene sequence that is identical to the HN gene of the HPIV-3 prototype strain isolated in 1957 in the United States (Figure 4). It is possible that such viruses have been continuously circulating over several years at a very low frequency and, so far, remained undetected or endemic in a single region. Currently, HPIV-3 cluster C is the most dynamic and widespread group worldwide [62], for which the emergence of new lineages and replacement of older lineages [2] have been described.

Limitations of the present study include limited sample sizes especially during the summer months, which is due to the structure of our surveillance system involving multiple sentinel practices. In addition, HPIV-2 and -4 sample sizes were somewhat lower compared to HPIV-1 and HPIV-3, which may affect observations on circulation patterns or associated clinical manifestations. With respect to study design, our sentinel prioritizes ARI patients with indications of systemic illness such as fever. This may lead to an underrepresentation of HPIV-4 infections, which are less frequently associated with fevers than other HPIV types [19,68]. Furthermore, our sentinel case definition of ILI includes cephalgias and myalgias, symptoms that are difficult to confirm in young children. Thus, the relatively low frequency of this symptom may in fact reflect the age distribution in our study cohort rather than true rarity. Finally, a portion of the HPIV-positive patients was coinfected with other respiratory viruses, and it is possible that in these individuals the clinical findings were due to the coinfecting pathogen, or the fact that more than one virus was involved, rather than HPIV alone. However, the overall findings on the clinical presentation of HPIV infection do line up well with those of other investigators, and the majority of cases examined here represent HPIV monoinfections, indicating that this limitation might not have a major effect.

## 5. Conclusions

In conclusion, HPIV infection is common among pediatric outpatients with acute respiratory infections in Germany. Our analyses confirm and expand knowledge on the seasonality of the different HPIV types, which appear to each follow biennial patterns that are clear for HPIV-1 and HPIV-2 and also present, albeit less pronounced, for HPIV-3 and HPIV-4. Furthermore, our data corroborate the notion that HPIV-3 is the most prevalent HPIV type and affects the youngest age groups, and that HPIV-4 infections are neither as rare nor as mild, as has been traditionally assumed. These findings are of particular interest with respect to future vaccine strategies, which should involve the administration of HPIV-3 vaccines at earlier ages than vaccines against the other HPIV types. Our study provides the first comprehensive molecular epidemiologic analysis of HPIV circulating in Germany, indicating for the first time the existence of two distinct genetic HPIV-2 groups. Despite the efforts of research groups worldwide, there is still a scarcity of molecular sequence information, resulting in a limited understanding of HPIV evolution. This poses substantial challenges (i) to conventional diagnostic systems, which may underdetect less common genetic variants, and (ii) to the development of effective vaccines that optimally prevent HPIV illness. In order to address these critical knowledge gaps, a global intensification of epidemiologic and molecular surveillance for respiratory pathogens in general and HPIV in particular is urgently needed.

## Figures and Tables

**Table 1:** Oligonucleotides for sequencing and differentiating HPIV 1–4.

Oligonucleotide	Polarity	Oligosequence (5’ → 3’)	TA ^1^ (°C)
External sequencing PCR			
HPIV1_Seq_6777_s	Sense	AGG GTT AAA GAC AAT CCA	55
HPIV1_Seq_8597_as	Antisense	TTT AAC ATA ATT GAT ATA G	55
HPIV2_Seq_6680_s	Sense	GGT TCA GTT CAA ATA TCG AC	53
HPIV2_Seq_8628_as	Antisense	GCT ATA ACA TTC TAA TAC AGC	53
HPIV3_Seq_6714_s	Sense	AAA GTT ACG CAA TCC AAC TC	55
HPIV3_Seq_8583_as	Antisense	CTC TTT TGT CTA TTG TCT GA	55
HPIV4a_Seq_7400_s	Sense	CAT AGA ATC TAA CAA TCC AGA	55
HPIV4a_Seq_9284_as	Antisense	ATT ACA GAA TGA TTT TGT TGA	55
HPIV4b_Seq_7454_s	Sense	AAT CTC CAA AAG AAA TAA CCC	55
HPIV4b_Seq_9394_as	Antisense	CCT GAT TAG TTC ATA GTG TTC	55
Nested sequencing PCR			
HPIV1_Seq_6777_s	Sense	AGG GTT AAA GAC AAT CCA	55
HPIV1_Seq_7450_as	Antisense	TGC ATA TAT TGC ATC ACC	55
HPIV1_Seq_7349_s	Sense	AAT ATC TCA TTA TTA CCT GGA C	55
HPIV1_Seq_8046_as	Antisense	TGA GTT ATT GGA ATA GTC TCG	55
HPIV1_Seq_7900_s	Sense	ATG TGC CAA TGT TAA TCA	55
HPIV1_Seq_8590as	Antisense	TTG ATA TAG TAT TAG GTC TTG ATC	55
HPIV2_Seq_6680_s	Sense	GGT TCA GTT CAA ATA TCG AC	57
HPIV2_Seq_7481_as	Antisense	TAA ATG GTT TTC ATA GTC CTG	57
HPIV2_Seq_7359_s	Sense	CAC TCA CAA TGT AAT ACT TGG	57
HPIV2_Seq_8024_as	Antisense	TTG TCA ATA ACA TAG AGC C	57
HPIV2_Seq_7893_s	Sense	AAA CAG GTT GAT TCA GAG TG	57
HPIV2_Seq_8592_as	Antisense	TCG CAT AAA ATA AAG AGC CT	57
HPIV3_Seq_6714_s	Sense	AAA GTT ACG CAA TCC AAC TC	57
HPIV3_Seq_7400_as	Antisense	GAG GTA TAA GCA TAA ATC AGA	57
HPIV3_Seq_7257_s	Sense	CAC GTC TGG TCT TCC ATC T	57
HPIV3_Seq_7929_as	Antisense	AAC AAT GAT GGA GTT GAC CAT	57
HPIV3_Seq_7785 s	Sense	TGG GTA TGG AGG TCT TGA AC	57
HPIV3_Seq_8577 as	Antisense	TGT CTA TTG TCT GAT TGC TGA TTA	57
HPIV4a_Seq_7400_s	Sense	CAT AGA ATC TAA CAA TCC AGA	55
HPIV4a_Seq_8067_as	Antisense	ACC TAA AGA GAA TGA TGG AA	55
HPIV4a_Seq_7921_s	Sense	TCA ACA AGT TCT ATT TCA CA	55
HPIV4a_Seq_8633_as	Antisense	TGT ATA AGT CTC CCT GAG A	55
HPIV4a_Seq_8456_s	Sense	ACA TTA TAT TTC CTT TGT ATG G	55
HPIV4a_Seq_9284_as	Antisense	ATT ACA GAA TGA TTT TGT TGA	55
HPIV4b_Seq_7454_s	Sense	AAT CTC CAA AAG AAA TAA CCC	55
HPIV4b_Seq_8132_as	Antisense	GAT ACA TAC TGT TTG CTA GAT	55
HPIV4b_Seq_7992_s	Sense	CAA TTT TAT TCC AAC TGC TAC	55
HPIV4b_Seq_8731_as	Antisense	TAT TAT ATA GGC GAC CCT CT	55
HPIV4b_Seq_8641_s	Sense	GAT TTA AGA CAA TTT CTT TCT G	55
HPIV4b_Seq_9394_as	Antisense	CCT GAT TAG TTC ATA GTG TTC	55
PCR differentiating between HPIV-4a and 4b			
HPIV_diff_2004_4a_s	Sense	GCT ACT ATA ATT TCT AAT TCC G	55
HPIV_diff_2210_4a_as	Antisense	CTT GAA ATC TTG GTT CCA CCA	55
HPIV_diff_16717_4b_s	Sense	AAA TGA ATG AGC AAG TAG TCG	55
HPIV_diff_16989_4b_as	Antisense	ATT TTA TTG TCT TTT GTC AGG	55

^1^ TA, Annealing temperature.

**Table 2:** Demographic information on specimens analyzed.

	Overall (%) *n* = 4463	2015/16 *n* = 896	2016/17 *n* = 1178	2017/18 *n* = 1279	2018/19 *n* = 1110
Gender					
Male	2363 (52.9)	473 (52.8)	628 (53.3)	699 (54.7)	563 (50.7)
Female	2081 (46.6)	419 (46.8)	548 (46.5)	573 (44.8)	541 (48.7)
Unknown	19 (0.4)	4 (0.4)	2 (0.2)	7 (0.5)	6 (0.5)
Age					
≤6 months	328 (7.3)	62 (6.9)	112 (9.5)	81 (6.3)	73 (6.6)
>6 months to ≤1 y	488 (10.9)	99 (11.0)	130 (11.0)	141 (11.0)	118 (10.6)
>1 y to ≤2 y	2303 (51.6)	442 (49.3)	614 (52.1)	670 (52.4)	577 (52.0)
>2 y to ≤4 y	1344 (30.1)	293 (32.7)	322 (27.3)	387 (30.3)	342 (30.8)

**Table 3:** Demographic information on HPIV-positive specimens.

	Overall (%) *n* = 459 (10.3% of Total)	2015/16 *n* = 75 (8.4%)	2016/17 *n* = 135 (11.5%)	2017/18 *n* = 119 (9.3%)	2018/19 *n* = 130 (11.7%)
Gender					
Male	250 (54.5)	40 (53.3)	76 (56.3)	61 (51.3)	73 (56.2)
Female	209 (45.5)	35 (46.7)	59 (43.7)	58 (48.7)	57 (43.8)
*Age*					
≤6 months	33 (7.2)	5 (6.7)	8 (5.9)	8 (6.7)	12 (9.2)
>6 months to ≤1 y	42 (9.2)	9 (12.0)	12 (8.9)	10 (8.4)	11 (8.5)
>1 y to ≤2 y	281 (61.2)	41 (54.7)	87 (64.4)	78 (65.5)	75 (57.7)
>2 y to ≤4 y	103 (22.4)	20 (26.7)	28 (20.7)	23 (19.3)	32 (24.6)

**Table 4:** Demographic and clinical data by HPIV-subtype.

	Total Samples (%) ^1^ *n* = 4463	HPIV 1–4 *n* = 459	HPIV-1 *n* = 113	HPIV-2 *n* = 49	HPIV-3 *n* =234	HPIV-4 *n* = 61	Coinfection ^2^ *n* = 2
Gender							
Male	2363 (52.9)	250 (54.5)	59 (52.2)	31 (63.3)	125 (53.4)	34 (55.7)	1 (50)
Female	2081 (46.6)	209 (45.5)	54 (47.8)	18 (36.7)	109 (46.6)	27 (44.3)	1 (50)
Unknown	19 (0.4)						
Median age in months (IQR) ^3^	24 (14–36)	22 (15–24)	**24** (19–36) **	24 (9–36)	**19** (14–24) **	22 (15–24)	24 (PIV2/3) 20 (PIV3/4)
Age category							
≤6 months	328 (7.3)	33 (7.2)	3 (2.7)	7 (14.3)	17 (7.3)	6 (9.8)	0
>6 months to ≤1 y	488 (10.9)	42 (9.2)	5 (4.4)	8 (16.3)	26 (11.1)	3 (4.9)	0
>1 y to ≤2 y	2303 (51.6)	281 (61.2)	62 (54.9)	15 (30.6)	161 (68.8)	41 (67.2)	2 (100)
>2 y to ≤4 y	1344 (30.1)	103 (22.4)	43 (38.1)	19 (38.8)	30 (12.8)	11 (18.0)	0
Clinical presentation ^4^							
Rhinorrhea	4034 (90.4)	**430** (93.7) *	102 (90.3)	46 (93.9)	**221** (94.4) *	59 (96.7)	2 (100)
Cough	3825 (85.7)	**418** (91.1) **	**105** (92.9) *	**48** (98.0) *	210 (89.7)	53 (86.9)	2 (100)
Fever/chills	4120 (92.3)	413 (90.0)	107 (94.7)	46 (93.9)	213 (91.0)	**45** (73.8) **	2 (100)
Pharyngitis	1851 (41.5)	186 (40.5)	54 (47.8)	22 (44.9)	90 (38.5)	18 (29.5)	2 (100)
Myalgias or cephalgias	1474 (33)	**114** (24.8) **	34 (30.1)	10 (20.4)	**54** (23.1) **	15 (24.6)	1 (50)
ILI	3603 (80.7)	376 (81.9)	99 (87.6)	43 (87.8)	195 (83.3)	**37** (60.7) **	2 (100)
Pneumonia	277 (6.2)	21 (4.6)	2 (1.8)	4 (8.2)	7 (3.0) *	**8** (13.1) *	0

^1^ This information, in part presented in Table 2, serves as a comparator for the HPIV-positive study population. For reasons of clarity, it is therefore relisted here. ^2^ This category includes one coinfection of HPIV-2 with HPIV-3 and one coinfection of HPIV-3 with HPIV-4. ^3^ denotes a highly significant age difference relative to the total study population (*p* < 0.005 using the nonparametric Mann–Whitney-U test) ^4^ Asterisks denote significant (* *p* < 0.05) or highly significant (** *p* < 0.005) differences in symptom prevalence relative to the total study population. *p*-values were calculated using the Chi-squared test.

**Table 5:** Distribution of German HPIV by Clade/Cluster and epidemic season ^1^.

	HPIV-1		HPIV-2	HPIV-3				HPIV-4	
Clade/Cluster	2	3	2.4	A	C1	C3	C5	2.1	2.2
2015/16	22	1			1	7	3		3
2016/17	5	13	13	1	2	13	12	2	
2017/18	4	5	1		1	15	11	1	
2018/19	1	6	14		1	20	10		
Total	32	25	28	1	5	55	36	3	3

^1^ A total of three viruses remained unclassified and are not listed in this table but are presented in the phylogenetic tree graphs in Figure 3 and Figure 4. One, from season 2015/16, belongs to HPIV type 3; one belongs to HPIV type 1 (2016/17); and one belongs to HPIV type 2 (2018/19).

## Data Availability

Not applicable.

## Supplementary Materials

The following are available online at https://www.mdpi.com/article/10.3390/microorganisms9071508/s1: Figure S1: Frequency of coinfections by viral type in HPIV-positive samples, Figure S2: Proportion of HPIV types 1-4 in all HPIV-positive samples, Figure S3: Maximum likelihood tree of partial HN gene of HPIV-1, Figure S4: Maximum likelihood tree of partial HN gene of HPIV-2, Figure S5: Maximum likelihood tree of partial HN gene of HPIV-4, Figure S6: Maximum likelihood tree of partial HN gene of HPIV-3, Table S1: Oligonucleotides used for the detection of HPIV 1–4.

## References

[1] Henrickson K.J. (2003). Parainfluenza viruses. Clin. Microbiol. Rev..

[2] Goya S., Mistchenko A.S., Viegas M. (2016). Phylogenetic and molecular analyses of human parainfluenza type 3 virus in Buenos Aires, Argentina, between 2009 and 2013: The emergence of new genetic lineages. Infect. Genet. Evol..

[3] Reed G., Jewett P.H., Thompson J., Tollefson S., Wright P.F. (1997). Epidemiology and clinical impact of parainfluenza virus infections in otherwise healthy infants and young children <5 years old. J. Infect. Dis..

[4] Pawelczyk M., Kowalski M.L. (2017). The Role of Human Parainfluenza Virus Infections in the Immunopathology of the Respiratory Tract. Curr. Allergy Asthma Rep..

[5] Schmidt A.C., Schaap-Nutt A., Bartlett E.J., Schomacker H., Boonyaratanakornkit J., Karron R.A., Collins P.L. (2011). Progress in the development of human parainfluenza virus vaccines. Expert Rev. Respir Med..

[6] Henrickson K.J., Savatski L.L. (1992). Genetic variation and evolution of human parainfluenza virus type 1 hemagglutinin neuraminidase: Analysis of 12 clinical isolates. J. Infect. Dis..

[7] Villaran M.V., Garcia J., Gomez J., Arango A.E., Gonzales M., Chicaiza W., Aleman W., Lorenzana de Rivera I., Sanchez F., Aguayo N. (2014). Human parainfluenza virus in patients with influenza-like illness from Central and South America during 2006–2010. Influenza Other Respir. Viruses.

[8] Mizuta K., Tsukagoshi H., Ikeda T., Aoki Y., Abiko C., Itagaki T., Nagano M., Noda M., Kimura H. (2014). Molecular evolution of the haemagglutinin-neuraminidase gene in human parainfluenza virus type 3 isolates from children with acute respiratory illness in Yamagata prefecture, Japan. J. Med. Microbiol..

[9] Mao N., Ji Y., Xie Z., Wang H., Wang H., An J., Zhang X., Zhang Y., Zhu Z., Cui A. (2012). Human parainfluenza virus-associated respiratory tract infection among children and genetic analysis of HPIV-3 strains in Beijing, China. PLoS ONE.

[10] Santak M., Lang Balija M., Mlinaric Galinovic G., Ljubin Sternak S., Vilibic-Cavlek T., Tabain I. (2018). Genotype replacement of the human parainfluenza virus type 2 in Croatia between 2011 and 2017—The role of neutralising antibodies. Epidemiol. Infect..

[11] Kosutic-Gulija T., Slovic A., Ljubin-Sternak S., Mlinaric-Galinovic G., Forcic D. (2017). Genetic analysis of human parainfluenza virus type 3 obtained in Croatia, 2011–2015. J. Med. Microbiol..

[12] Rima B., Balkema-Buschmann A., Dundon W.G., Duprex P., Easton A., Fouchier R., Kurath G., Lamb R., Lee B., Rota P. (2019). ICTV Virus Taxonomy Profile: Paramyxoviridae. J. Gen. Virol..

[13] Alvarez-Arguelles M.E., Rojo-Alba S., Perez Martinez Z., Leal Negredo A., Boga Riveiro J.A., Alonso Alvarez M.A., Rodriguez Suarez J., de Ona Navarro M., Melon Garcia S. (2018). New clinical and seasonal evidence of infections by Human Parainfluenzavirus. Eur. J. Clin. Microbiol. Infect. Dis..

[14] Aguilar J.C., Perez-Brena M.P., Garcia M.L., Cruz N., Erdman D.D., Echevarria J.E. (2000). Detection and identification of human parainfluenza viruses 1, 2, 3, and 4 in clinical samples of pediatric patients by multiplex reverse transcription-PCR. J. Clin. Microbiol..

[15] Knott A.M., Long C.E., Hall C.B. (1994). Parainfluenza viral infections in pediatric outpatients: Seasonal patterns and clinical characteristics. Pediatr. Infect. Dis. J..

[16] Henrickson K.J., Hoover S., Kehl K.S., Hua W. (2004). National disease burden of respiratory viruses detected in children by polymerase chain reaction. Pediatr. Infect. Dis. J..

[17] Weinberg G.A., Hall C.B., Iwane M.K., Poehling K.A., Edwards K.M., Griffin M.R., Staat M.A., Curns A.T., Erdman D.D., Szilagyi P.G. (2009). Parainfluenza virus infection of young children: Estimates of the population-based burden of hospitalization. J. Pediatr..

[18] Branche A.R., Falsey A.R. (2016). Parainfluenza Virus Infection. Semin Respir Crit Care Med..

[19] Rubin E.E., Quennec P., McDonald J.C. (1993). Infections due to parainfluenza virus type 4 in children. Clin. Infect. Dis..

[20] Frost H.M., Robinson C.C., Dominguez S.R. (2014). Epidemiology and clinical presentation of parainfluenza type 4 in children: A 3-year comparative study to parainfluenza types 1–3. J. Infect. Dis..

[21] Lau S.K., Li K.S., Chau K.Y., So L.Y., Lee R.A., Lau Y.L., Chan K.H., Lim W.W., Woo P.C., Yuen K.Y. (2009). Clinical and molecular epidemiology of human parainfluenza virus 4 infections in hong kong: Subtype 4B as common as subtype 4A. J. Clin. Microbiol..

[22] Fry A.M., Curns A.T., Harbour K., Hutwagner L., Holman R.C., Anderson L.J. (2006). Seasonal trends of human parainfluenza viral infections: United States, 1990–2004. Clin. Infect. Dis..

[23] Yano T., Fukuta M., Maeda C., Akachi S., Matsuno Y., Yamadera M., Kobayashi A., Nagai Y., Kusuhara H., Kobayashi T. (2014). Epidemiological investigation and seroprevalence of human parainfluenza virus in Mie Prefecture in Japan during 2009–2013. Jpn. J. Infect. Dis..

[24] Glezen W.P., Frank A.L., Taber L.H., Kasel J.A. (1984). Parainfluenza virus type 3: Seasonality and risk of infection and reinfection in young children. J. Infect. Dis..

[25] Vesa S., Kleemola M., Blomqvist S., Takala A., Kilpi T., Hovi T. (2001). Epidemiology of documented viral respiratory infections and acute otitis media in a cohort of children followed from two to twenty-four months of age. Pediatr. Infect. Dis. J..

[26] Henrickson K.J., Kuhn S.M., Savatski L.L. (1994). Epidemiology and cost of infection with human parainfluenza virus types 1 and 2 in young children. Clin. Infect. Dis..

[27] Glezen W.P., Greenberg S.B., Atmar R.L., Piedra P.A., Couch R.B. (2000). Impact of respiratory virus infections on persons with chronic underlying conditions. JAMA.

[28] Greenberg S.B., Allen M., Wilson J., Atmar R.L. (2000). Respiratory viral infections in adults with and without chronic obstructive pulmonary disease. Am. J. Respir. Crit Care Med..

[29] Garg R., Brownlie R., Latimer L., Gerdts V., Potter A., van Drunen Littel-van den Hurk S. (2018). A chimeric glycoprotein formulated with a combination adjuvant induces protective immunity against both human respiratory syncytial virus and parainfluenza virus type 3. Antivir. Res..

[30] Karron R.A., Belshe R.B., Wright P.F., Thumar B., Burns B., Newman F., Cannon J.C., Thompson J., Tsai T., Paschalis M. (2003). A live human parainfluenza type 3 virus vaccine is attenuated and immunogenic in young infants. Pediatr. Infect. Dis. J..

[31] Gomez M., Mufson M.A., Dubovsky F., Knightly C., Zeng W., Losonsky G. (2009). Phase-I study MEDI-534, of a live, attenuated intranasal vaccine against respiratory syncytial virus and parainfluenza-3 virus in seropositive children. Pediatr. Infect. Dis. J..

[32] Shaw C., Lee H., Knightly C., Kalidindi S., Zaks T., Smolenov I., Panther L. (2019). 2754. Phase 1 Trial of an mRNA-Based Combination Vaccine Against hMPV and PIV3. Open Forum Infect. Dis..

[33] Wu K., Choi A., Koch M., Ma L., Hill A., Nunna N., Huang W., Oestreicher J., Colpitts T., Bennett H. (2021). Preliminary Analysis of Safety and Immunogenicity of a SARS-CoV-2 Variant Vaccine Booster. Medrxiv.

[34] Wu K., Choi A., Koch M., Elbashir S., Ma L., Lee D., Woods A., Henry C., Palandjian C., Hill A. (2021). Variant SARS-CoV-2 mRNA vaccines confer broad neutralization as primary or booster series in mice. bioRxiv.

[35] Jornist I., Muhsen K., Ram D., Lustig Y., Levy V., Orzitser S., Azar R., Weil M., Indenbaum V., Sofer D. (2018). Characterization of human parainfluenza virus-3 circulating in Israel, 2012–2015. J. Clin. Virol..

[36] Takahashi M., Nagasawa K., Saito K., Maisawa S.I., Fujita K., Murakami K., Kuroda M., Ryo A., Kimura H. (2018). Detailed genetic analyses of the HN gene in human respirovirus 3 detected in children with acute respiratory illness in the Iwate Prefecture, Japan. Infect. Genet. Evol..

[37] Elusah J., Bulimo W.D., Opanda S.M., Symekher S.L., Wamunyokoli F. (2020). Genetic diversity and evolutionary analysis of human respirovirus type 3 strains isolated in Kenya using complete hemagglutinin-neuraminidase (HN) gene. PLoS ONE.

[38] Almajhdi F.N., Alshaman M.S., Amer H.M. (2012). Human parainfluenza virus type 2 hemagglutinin-neuramindase gene: Sequence and phylogenetic analysis of the Saudi strain Riyadh 105/2009. Virol. J..

[39] Santak M., Slovic A., Ljubin-Sternak S., Mlinaric Galinovic G., Forcic D. (2016). Genetic diversity among human parainfluenza virus type 2 isolated in Croatia between 2011 and 2014. J. Med. Virol..

[40] Kiptinness J.K., Wurapa E.K., Wamunyokoli F., Bulimo W.D. (2013). Molecular characterization of human parainfluenza virus type 1 in infants attending Mbagathi District Hospital, Nairobi, Kenya: A retrospective study. Virus Genes.

[41] Beck E.T., He J., Nelson M.I., Bose M.E., Fan J., Kumar S., Henrickson K.J. (2012). Genome sequencing and phylogenetic analysis of 39 human parainfluenza virus type 1 strains isolated from 1997–2010. PLoS ONE.

[42] Obodai E., Odoom J.K., Adiku T., Goka B., Wolff T., Biere B., Schweiger B., Reiche J. (2018). The significance of human respiratory syncytial virus (HRSV) in children from Ghana with acute lower respiratory tract infection: A molecular epidemiological analysis, 2006 and 2013–2014. PLoS ONE.

[43] Reiche J., Jacobsen S., Neubauer K., Hafemann S., Nitsche A., Milde J., Wolff T., Schweiger B. (2014). Human metapneumovirus: Insights from a ten-year molecular and epidemiological analysis in Germany. PLoS ONE.

[44] Reiche J., Schweiger B. (2009). Genetic variability of group A human respiratory syncytial virus strains circulating in Germany from 1998 to 2007. J. Clin. Microbiol..

[45] Oh D.-Y., Buda S., Biere B., Schlosser F., Duwe S., Wedde M., von Kleist M., Mielke M., Wolff T., Durrwald R. (2021). Trends in respiratory virus circulation following COVID-19-targeted nonpharmaceutical interventions in Germany, January–September 2020: Analysis of national surveillance data. Lancet Reg. Health Eur..

[46] Fritsch A., Schweiger B., Biere B. (2019). Influenza C virus in pre-school children with respiratory infections: Retrospective analysis of data from the national influenza surveillance system in Germany, 2012 to 2014. Eurosurveillance.

[47] ICTV Paramyxoviridae Study Group (2016). Implementation of taxon-wide non-Latinized binomial species names in the family Paramyxoviridae.

[48] ICTV Paramyxoviridae Study Group (2018). Re-organization of the family Paramyxoviridae.

[49] Chmielewicz B., Nitsche A., Schweiger B., Ellerbrok H. (2005). Development of a PCR-Based Assay for Detection, Quantification, and Genotyping of Human Adenoviruses. Clin. Chem..

[50] Katoh K., Misawa K., Kuma K., Miyata T. (2002). MAFFT: A novel method for rapid multiple sequence alignment based on fast Fourier transform. Nucleic Acids Res..

[51] Katoh K., Standley D.M. (2013). MAFFT multiple sequence alignment software version 7: Improvements in performance and usability. Mol. Biol. Evol..

[52] Kumar S., Stecher G., Li M., Knyaz C., Tamura K. (2018). MEGA X: Molecular Evolutionary Genetics Analysis across Computing Platforms. Mol. Biol. Evol..

[53] Linster M., Do L.A.H., Minh N.N.Q., Chen Y., Zhe Z., Tuan T.A., Tuan H.M., Su Y.C.F., van Doorn H.R., Moorthy M. (2018). Clinical and Molecular Epidemiology of Human Parainfluenza Viruses 1–4 in Children from Viet Nam. Sci. Rep..

[54] Steffens A., Finelli L., Whitaker B., Fowlkes A. (2016). Population-based Surveillance for Medically Attended Human Parainfluenza Viruses From the Influenza Incidence Surveillance Project, 2010–2014. Pediatr. Infect. Dis. J..

[55] Shi W., Cui S., Gong C., Zhang T., Yu X., Li A., Chen M., Luo M., Huang F. (2015). Prevalence of human parainfluenza virus in patients with acute respiratory tract infections in Beijing, 2011–2014. Influenza Other Respir. Viruses.

[56] Bellau-Pujol S., Vabret A., Legrand L., Dina J., Gouarin S., Petitjean-Lecherbonnier J., Pozzetto B., Ginevra C., Freymuth F. (2005). Development of three multiplex RT-PCR assays for the detection of 12 respiratory RNA viruses. J. Virol. Methods.

[57] Templeton K.E., Bredius R.G., Scheltinga S.A., Claas E.C., Vossen J.M., Kroes A.C. (2004). Parainfluenza virus 3 infection pre- and post-haematopoietic stem cell transplantation: Re-infection or persistence?. J. Clin. Virol..

[58] Templeton K.E., Bredius R.G., Claas E.C., Kroes A.C., Walther F.J. (2005). Parainfluenza virus 4 detection in infants. Eur. J. Pediatr..

[59] Esposito S., Daleno C., Prunotto G., Scala A., Tagliabue C., Borzani I., Fossali E., Pelucchi C., Principi N. (2012). Impact of viral infections in children with community-acquired pneumonia: Results of a study of 17 respiratory viruses. Influenza Other Respir. Viruses.

[60] Emanuels A., Newman K.L., Hawes S.E., Martin E.T., A Englund J., Tielsch J., Kuypers J., Katz J., Khatry S., LeClerq S. (2019). Respiratory Viral Coinfection in a Birth Cohort of Infants in Rural Nepal. Open Forum Infect. Dis..

[61] Ruampunpong H., Payungporn S., Samransamruajkit R., Pratheepamornkul T., Theamboonlers A., Poovorawan Y. (2014). Human parainfluenza virus infection in Thai children with lower respiratory tract infection from 2010 to 2013. Southeast Asian J. Trop. Med. Public Health.

[62] Pan Y., Zhang Y., Shi W., Peng X., Cui S., Zhang D., Lu G., Liu Y., Wu S., Yang P. (2017). Human parainfluenza virus infection in severe acute respiratory infection cases in Beijing, 2014-2016: A molecular epidemiological study. Influenza Other Respir. Viruses.

[63] Howard L.M., Rankin D.A., Spieker A.J., Gu W., Haddadin Z., Probst V., Rahman H., McHenry R., Pulido C.G., Williams J.V. (2021). Clinical features of parainfluenza infections among young children hospitalized for acute respiratory illness in Amman, Jordan. BMC Infect. Dis..

[64] Vachon M.L., Dionne N., Leblanc E., Moisan D., Bergeron M.G., Boivin G. (2006). Human parainfluenza type 4 infections, Canada. Emerg. Infect. Dis..

[65] Hall C.B. (2001). Respiratory syncytial virus and parainfluenza virus. N. Engl. J. Med..

[66] Liu W.K., Liu Q., Chen D.H., Liang H.X., Chen X.K., Huang W.B., Qin S., Yang Z.F., Zhou R. (2013). Epidemiology and clinical presentation of the four human parainfluenza virus types. BMC Infect. Dis..

[67] Fox T.G., Christenson J.C. (2014). Influenza and parainfluenza viral infections in children. Pediatr. Rev..

[68] Lau S.K., To W.K., Tse P.W., Chan A.K., Woo P.C., Tsoi H.W., Leung A.F., Li K.S., Chan P.K., Lim W.W. (2005). Human parainfluenza virus 4 outbreak and the role of diagnostic tests. J. Clin. Microbiol..

[69] Murphy B., Phelan P.D., Jack I., Uren E. (1980). Seasonal pattern in childhood viral lower respiratory tract infections in Melbourne. Med. J. Aust..

[70] Carballal G., Videla C.M., Espinosa M.A., Savy V., Uez O., Sequeira M.D., Knez V., Requeijo P.V., Posse C.R., Miceli I. (2001). Multicentered study of viral acute lower respiratory infections in children from four cities of Argentina, 1993–1994. J. Med. Virol..

[71] Marx A., Torok T.J., Holman R.C., Clarke M.J., Anderson L.J. (1997). Pediatric hospitalizations for croup (laryngotracheobronchitis): Biennial increases associated with human parainfluenza virus 1 epidemics. J. Infect. Dis..

[72] Takashita E., Kawakami C., Momoki T., Saikusa M., Shimizu K., Ozawa H., Kumazaki M., Usuku S., Tanaka N., Okubo I. (2021). Increased risk of rhinovirus infection in children during the coronavirus disease-19 pandemic. Influenza Other Respir. Viruses.

[73] Foley D.A., Yeoh D.K., Minney-Smith C.A., Martin A.C., Mace A.O., Sikazwe C.T., Le H., Levy A., Moore H.C., Blyth C.C. (2021). The Interseasonal Resurgence of Respiratory Syncytial Virus in Australian Children Following the Reduction of Coronavirus Disease 2019-Related Public Health Measures. Clin. Infect. Dis..

[74] Bose M.E., Shrivastava S., He J., Nelson M.I., Bera J., Fedorova N., Halpin R., Town C.D., Lorenzi H.A., Amedeo P. (2019). Sequencing and analysis of globally obtained human parainfluenza viruses 1 and 3 genomes. PLoS ONE.

[75] Godoy C., Peremiquel-Trillas P., Andres C., Gimferrer L., Uriona S.M., Codina M.G., Armadans L., Martin Mdel C., Fuentes F., Esperalba J. (2016). A molecular epidemiological study of human parainfluenza virus type 3 at a tertiary university hospital during 2013–2015 in Catalonia, Spain. Diagn. Microbiol. Infect. Dis..

[76] Kosutic-Gulija T., Slovic A., Ljubin-Sternak S., Mlinaric-Galinovic G., Forcic D. (2016). A study of genetic variability of human parainfluenza virus type 1 in Croatia, 2011–2014. J. Med. Microbiol..

[77] Terrier O., Cartet G., Ferraris O., Morfin F., Thouvenot D., Hong S.S., Lina B. (2008). Characterization of naturally occurring parainfluenza virus type 2 (hPIV-2) variants. J. Clin. Virol..

[78] Tsurudome M., Nishio M., Komada H., Bando H., Ito Y. (1989). Extensive antigenic diversity among human parainfluenza type 2 virus isolates and immunological relationships among paramyxoviruses revealed by monoclonal antibodies. Virology.

[79] Numazaki Y., Shigeta S., Yano N., Takai S., Ishida N. (1968). A variant of parainfluenza type 2 virus. Proc. Soc. Exp. Biol. Med..

[80] Canchola J., Vargosko A.J., Kim H.W., Parrott R.H., Christmas E., Jeffries B., Chanock R.M. (1964). Antigenic Variation among Newly Isolated Strains of Parainfluenza Type 4 Virus. Am. J. Hyg..

