# Supplementary material for: Virological Surveillance and Molecular Characterization of Human Parainfluenzavirus Infection in Children with Acute Respiratory Illness: Germany, 2015–2019
Source: Microorganisms. 2021 Jul 14;9(7):1508. doi: 10.3390/microorganisms9071508 (PMC8307145; doi:10.3390/microorganisms9071508)
Supplement: Supplementary file 1 [file microorganisms-09-01508-s001.zip › Figure_S2_Oh_et_al.pdf]

Virological surveillance and Molecular Characterization of Human Parainfluenzavirus Infection in Children with Acute Respiratory Illness: Germany, 2015-2019

Djin-Ye Oh, Barbara Biere, Markus Grenz, Thorsten Wolff, Brunhilde Schweiger, Ralf Dürrwald, Janine Reiche

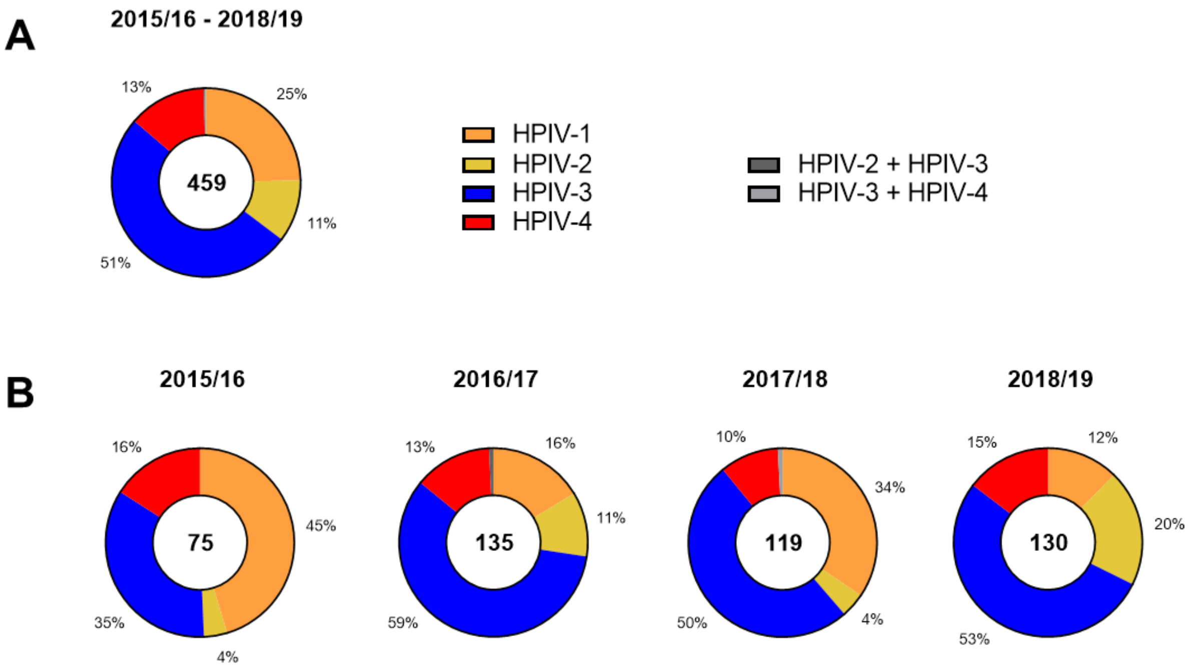

Figure S2: Proportion of HPIV types 1-4 in all HPIV-positive samples. (A) overall and (B) per season. Percentage values indicated for each HPIV type refer to the total number of HPIV-positive samples, which is provided in the center of each piechart.
