# Supplementary material for: Virological Surveillance and Molecular Characterization of Human Parainfluenzavirus Infection in Children with Acute Respiratory Illness: Germany, 2015–2019
Source: Microorganisms. 2021 Jul 14;9(7):1508. doi: 10.3390/microorganisms9071508 (PMC8307145; doi:10.3390/microorganisms9071508)
Supplement: Supplementary file 1 [file microorganisms-09-01508-s001.zip › Figure_S5_Oh_et_al.pdf]

Virological Surveillance and Molecular Characterization of Human Parainfluenzavirus Infection in Children with Acute Respiratory Illness: Germany, 2015-2019

Djin-Ye Oh, Barbara Biere, Markus Grenz, Thorsten Wolff, Brunhilde Schweiger, Ralf Dürrwald, Janine Reiche

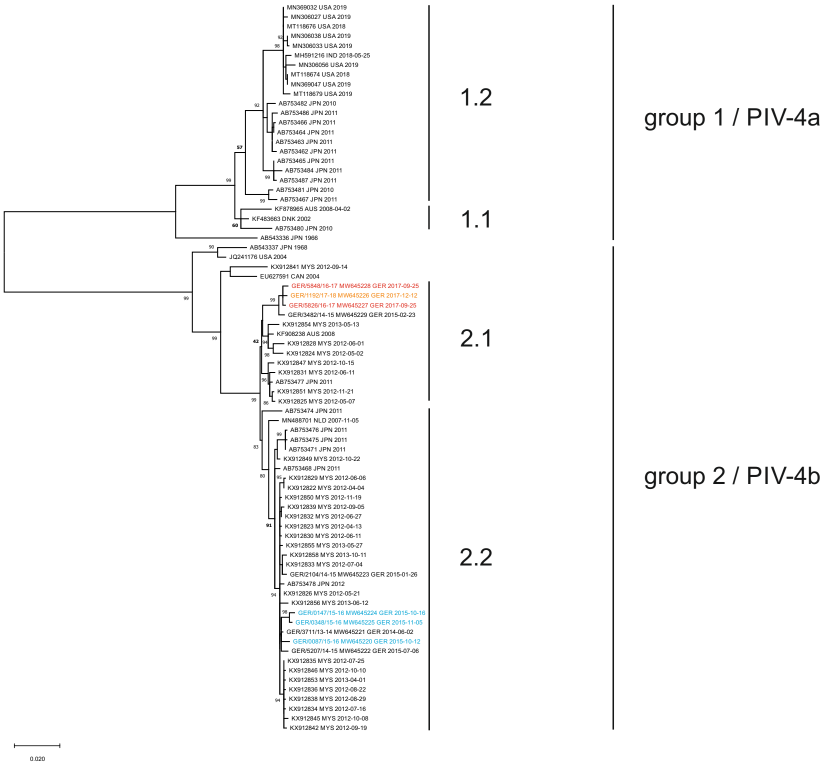

Figure S5: Maximum likelihood tree of partial HN gene of HPIV-4. German sequences of this study are coloured by epidemic season: 2015/16 in blue, 2016/17 in red, 2017/18 in orange, and 2018/19 in green. Clades are indicated on the right. Only bootstrap values greater or equal 80% are displayed at the branch nodes.
