# Supplementary material for: Virological Surveillance and Molecular Characterization of Human Parainfluenzavirus Infection in Children with Acute Respiratory Illness: Germany, 2015–2019
Source: Microorganisms. 2021 Jul 14;9(7):1508. doi: 10.3390/microorganisms9071508 (PMC8307145; doi:10.3390/microorganisms9071508)
Supplement: Supplementary file 1 [file microorganisms-09-01508-s001.zip › Table_S1_Oh_et_al.pdf]

Virological surveillance and Molecular Characterization of Human Parainfluenzavirus Infection in Children with Acute Respiratory Illness: Germany, 2015-2019

Djin-Ye Oh, Barbara Biere, Markus Grenz, Thorsten Wolff, Brunhilde Schweiger, Ralf Dürrwald, Janine Reiche

The following table provides oligonucleotide sequences used in the multiplex real-time RT-PCR for the detection of HPIV-1-4 as described previously [45].

Table S1: Oligonucleotides used for the detection of HPIV 1-4

PCR	Oligonucleotide	Sequence (5′→3′)	Final concentration
PIV-1 [HN-Gene]	HPIV-1 F7508	TGCAATATATGCRTATTCATCAAACTTAAT	300
	HPIV-1 R7587	CTAATTGTAAAACCTGATATGACTTCCCTA	300
	HPIV-1 MGB7539	*ACTCAAGGATGTGCAGATA - MGB*	100
PIV-2 [HN-Gene]	HPIV-2 F7460	ATCTTCAGGACTATGAAAACCATTTACC	300
	HPIV-2 R7544	CACAACCTCCTGGTATAGCAGTGAC	300
	HPIV-2 TM7489	*AAGTGATGGAATCAATCGCAAAAGCTGTT*	100
PIV-3 [HN-Gene]	HPIV-3 F8271	GCATTGTATCATCTGTCATATTRGAYTCAC	600
	HPIV-3 R8364	GCCAGCTCGTTYACYCTTTCRGT	600
	HPIV-3 TM8306	*TCGAGAGTBAACCCAGTCATAACTTACTCAACA*	150
PIV-4 [P-Gene]	HPIV-4 F3028	AGACGTCTCAAAATTTGTTGATCAAG	300
	HPIV-4 R3103	GGTTCCAGAYAAWATGGGTCTTGCTA	600
	HPIV-4 MGB3085	*TCAAGTGTAATTGTATTRTC - MGB*	150
